# Systemic therapy with or without transcatheter intra-arterial therapies for unresectable hepatocellular carcinoma: a real-world, multi-center study

**DOI:** 10.3389/fimmu.2023.1138355

**Published:** 2023-04-26

**Authors:** Yangxun Pan, Xiaodong Zhu, Jianwei Liu, Jianhong Zhong, Wei Zhang, Shunli Shen, Renan Jin, Hongzhi Liu, Feng Ye, Kuan Hu, Da Xu, Yu Zhang, Zhong Chen, Baocai Xing, Ledu Zhou, Yongjun Chen, Yongyi Zeng, Xiao Liang, Ming Kuang, Tianqiang Song, Bangde Xiang, Kui Wang, Huichuan Sun, Li Xu, China Liver Cancer Study Group Young Investigators (CLEAP)

**Affiliations:** ^1^ Department of Liver Surgery, Sun Yat-sen University Cancer Center, Sun Yat-sen University, Guangzhou, China; ^2^ State Key Laboratory of Oncology in South China, Collaborative Innovation Center for Cancer Medicine, Guangzhou, China; ^3^ Department of Liver Surgery and Transplantation, Liver Cancer Institute and Zhongshan Hospital, Fudan University, Shanghai, China; ^4^ Department of Hepatic Surgery II, The Eastern Hepatobiliary Surgery Hospital, Naval Medical University, Shanghai, China; ^5^ Department of Hepatobiliary Surgery, Guangxi Medical University Cancer Hospital, Guangxi Medical University, Nanning, China; ^6^ Department of Hepatobiliary Surgery, Tianjin Medical University Cancer Institute and Hospital, Tianjin Medical University, Tianjin, China; ^7^ Department of Hepatic Surgery, The First Affiliated Hospital of Sun Yat-sen University, Sun Yat-sen University, Guangzhou, China; ^8^ Department of General Surgery, Sir Run Run Shaw Hospital, College of Medicine, Institute of Minimally Invasive Surgery, Zhejiang University, Hangzhou, China; ^9^ Department of Hepatobiliary Surgery, Mengchao Hepatobiliary Hospital of Fujian Medical University, Fujian Medical University, Fuzhou, China; ^10^ Department of General Surgery, Ruijin Hospital, Shanghai Jiao Tong University School of Medicine, Shanghai, China; ^11^ Department of Hepatobiliary Surgery, Xiangya Hospital, Central South University, Changsha, China; ^12^ Key Laboratory of Carcinogenesis and Translational Research (Ministry of Education/Beijing), Hepatopancreatobiliary Surgery Department I, Peking University Cancer Hospital and Institute, Beijing, China; ^13^ Department of Hepatobiliary and Pancreatic Surgery, Affiliated Hospital of Nantong University, Nantong, China

**Keywords:** hepatocellular carcinoma, transcatheter intra-arterial therapies, systemic therapy, combination therapy, prognosis

## Abstract

**Background:**

Systemic therapy is the standard care of unresectable hepatocellular carcinoma (uHCC), while transcatheter intra-arterial therapies (TRITs) were also widely applied to uHCC patients in Chinese practice. However, the benefit of additional TRIT in these patients is unclear. This study investigated the survival benefit of concurrent TRIT and systemic therapy used as first-line treatment for patients with uHCC.

**Methods:**

This real-world, multi-center retrospective study included consecutive patients treated at 11 centers accross China between September 2018 and April 2022. Eligible patients had uHCC of China liver cancer stages IIb to IIIb (Barcelona clinic liver cancer B or C stage), and received first-line systemic therapy with or without concurrent TRIT. Of 289 patients included, 146 received combination therapy and 143 received systemic therapy alone. The overall survival (OS), as primary outcomes, was compared between patients who received systemic therapy plus TRIT (combination group) or systemic therapy alone (systemic-only group) using survival analysis and Cox regression. Imbalances in baseline clinical features between the two groups were adjusted through propensity score matching (PSM) and inverse probability of treatment weighting (IPTW). Moreover, subgroup analysis was conducted based on the different tumor characteristics of enrolled uHCC patients.

**Results:**

The median OS was significantly longer in the combination group than the systemic-only group before adjustment [not reached *vs.* 23.9 months; hazard ratio (HR), 0.561; 95% confidence interval (CI), 0.366 to 0.861; *P* = 0.008], after PSM (HR, 0.612; 95% CI, 0.390 to 0.958; *P* = 0.031) and after IPTW (HR, 0.539; 95% CI, 0.116 to 0.961; *P* = 0.008). Subgroup analyses suggested the benefit of combining TRIT with systemic therapy was greatest in patients with liver tumors exceeding the up-to-seven criteria, with an absence of extrahepatic metastasis, or with alfa-fetoprotein ≥ 400 ng/ml.

**Conclusion:**

Concurrent TRIT with systemic therapy was associated with improved survival compared with systemic therapy alone as first-line treatment for uHCC, especially for patients with high-intrahepatic tumor load and no extrahepatic metastasis.

## Introduction

Primary liver cancer was the sixth most common malignancy and third leading cause of cancer death worldwide in 2020, with hepatocellular carcinoma (HCC) accounting for 75 to 85% of cases (1). Since HCC generally progresses asymptomatically, most patients in China are diagnosed with intermediate- or advanced-stage disease, which is not amenable to radical therapies and has a poor long-term prognosis (2). Systemic therapies, including tyrosine kinase inhibitors (TKIs), bevacizumab, and immune checkpoint inhibitors (ICIs), are recommended as first-line treatments for patients with advanced- or intermediate-stage HCC with diffuse or extensive liver involvement (3). Chinese treatment guidelines for HCC also recommend transarterial chemoembolization (TACE) for patients with unresectable hepatocellular carcinoma (uHCC) (4). In clinical practice, many patients with uHCC receive TACE or other transcatheter intra-arterial therapies (TRITs), such as hepatic artery infusion chemotherapy (HAIC), in combination with systemic therapy. However, whether the addition of TRIT improves outcomes compared with systemic therapy alone remains controversial.

Significant advances in locoregional and systemic therapies in recent years have substantially improved the prognosis for patients with uHCC. For example, HAIC with oxaliplatin, fluorouracil, and leucovorin (FOLFOX-HAIC) has emerged as a more potent TRIT compared with TACE for patients with tumors larger than 7 cm (5). However, despite decades of development, only two TKIs, sorafenib and lenvatinib, are currently recommended as first-line systemic therapy for patients with advanced HCC, and these agents provide only a limited benefit (6, 7). Nonetheless, highly promising preliminary results from the IMbrave150 and LEAP-002 trials showing objective response rates (ORRs) of 30% or higher have provided support for the further investigation of combinations of molecularly targeted drugs (MTDs) and ICIs for HCC treatment (8, 9).

Given the trend toward an increasing use of combination therapies for uHCC, there is significant interest in the potential of systemic therapy plus TRIT to improve tumor control (10, 11). Of note, sorafenib plus FOLFOX-HAIC or lenvatinib plus TACE have recently been shown to prolong median overall survival (OS) by 5 to 6 months compared with TKIs alone (12, 13). Several small, exploratory trials of TRIT combined with MTDs plus ICIs have also shown encouraging efficacy in patients with uHCC (14–18) but were mostly limited by single-arm, single-center designs. Overall, there is a lack of data from multi-center, head-to-head studies comparing outcomes between MTD plus ICI combinations, when administered with or without TRIT.

In the present study, we compared long-term outcomes for patients receiving dual (MTD plus ICI) systemic therapy, with or without TACE/HAIC, using the large-scale China Liver Cancer Study Group Young Investigators (CLEAP) database, which includes data from 34 clinical centers across China. The analysis was expected to provide preliminary evidence that will inform the future use of these treatment strategies in patients with uHCC.

## Materials and methods

### Patients

The CLEAP database contains data from 843 patients diagnosed with uHCC between September 2018 and April 2022 and treated at 34 clinical centers across China. Inclusion criteria for the present analysis were as follows: diagnosis of HCC according to clinical or pathological features based on the American Association for the Study of Liver Diseases practice guidelines (19); China liver cancer (CNLC) stage IIb, IIIa or IIIb (4), which corresponds to Barcelona Clinic liver cancer stage B or C; receipt of first-line systemic therapy with TKIs plus ICIs, with or without concurrent TRIT; Eastern Cooperative Oncology Group performance status (ECOG PS) 0 or 1 (20); and availability of complete medical and follow-up data. Exclusion criteria were as follows: diagnosis of other malignant tumors; use of local therapies other than TACE or HAIC; and use of systemic therapy regimens other than combinations of TKIs plus anti-programmed death-1 (PD-1) antibodies. In addition, patients from centers that contributed fewer than five cases were excluded to avoid any potential impact of limited clinical experience with these regimens. Eligible patients were analyzed according to whether they received first-line systemic therapy with concurrent TRIT (combination group) or without concurrent TRIT (systemic-only group). Patients who received TRIT after confirmed progressive disease (PD) on systemic therapy were included in the systemic-only group. Systemic agents were administered according to the product package inserts and previous studies, based on current efficacy and safety data, prior treatment, and drug access (21).

The protocol complied with the ethical guidelines of the Declaration of Helsinki of the World Medical Association and was approved by the Institutional Review Boards of Sun Yat-sen University Cancer Center and Zhongshan Hospital, Fudan University (approval nos. B2022-301-01 and B20202-195R, respectively). All patients provided written informed consent for HCC treatment and the use of their medical records for research purposes.

### Clinical assessments

Tumor assessments were performed every two to three cycles (i.e., every 6 to 12 weeks) using contrast enhanced computed tomography (CT) or magnetic resonance imaging for intrahepatic tumors and upper abdominal metastasis and chest CT for lung metastases, with blood tests used to evaluate liver function and tumor markers. Tumor responses were defined as complete response (CR), partial response (PR), stable disease (SD), or PD according to the Response Evaluation Criteria in Solid Tumors (RECIST) v1.1 (22). The ORR was calculated as the proportion of patients with the best response of CR or PR. OS was defined as the time from treatment initiation to cancer-related death. Progression-free survival (PFS) was defined as the time from treatment initiation to disease progression or death from any cause. Safety was evaluated based on the occurrence of severe (i.e., grade ≥ 3) treatment-related adverse events (TRAEs).

### Statistical analysis

Continuous variables were summarized as means [standard deviation (SD)] or medians [interquartile range (IQR)], while categorical variables were summarized as counts (percentage). The Student’s t-test and Mann–Whitney *U* test were used to compare normally and non-normally distributed continuous variables, respectively. Categorical variables were compared using the chi-square or Fisher’s exact test, as appropriate. Survival analyses were performed using the Kaplan–Meier method, with comparison of OS and PFS curves conducted using the log-rank test. Survival outcomes were also analyzed by Cox regression using both univariate and multivariate models including relevant variables. Subgroup analyses were performed according to extrahepatic metastasis (presence or absence), intrahepatic tumor burden (within or exceeding the up-to-seven criteria) (23), alfa-fetoprotein (AFP) levels (< 400 or ≥ 400 ng/ml), and type of TRIT received (TACE or HAIC).

To adjust for differences in potential confounding variables, propensity scores were calculated using logistic regression and matched for 1:1 nearest-neighbor individuals according to the logit of the propensity scores, with a caliper width of 0.1 times the SD of the propensity score logit (24). Propensity score matching (PSM) was performed with the “MatchIt” package within R software and included the variables tumor size, hepatic vein invasion and ECOG PS. In addition, inverse probability of treatment weighting (IPTW) was performed as a sensitivity analysis with the same variables using the “WeightIt” package within R (25). All statistical analyses were performed using R software version 4.2.1 (http://www.r-project.org/). *P*-values of < 0.05 were considered statistically significant.

## Results

### Baseline characteristics

**Figure 1:**
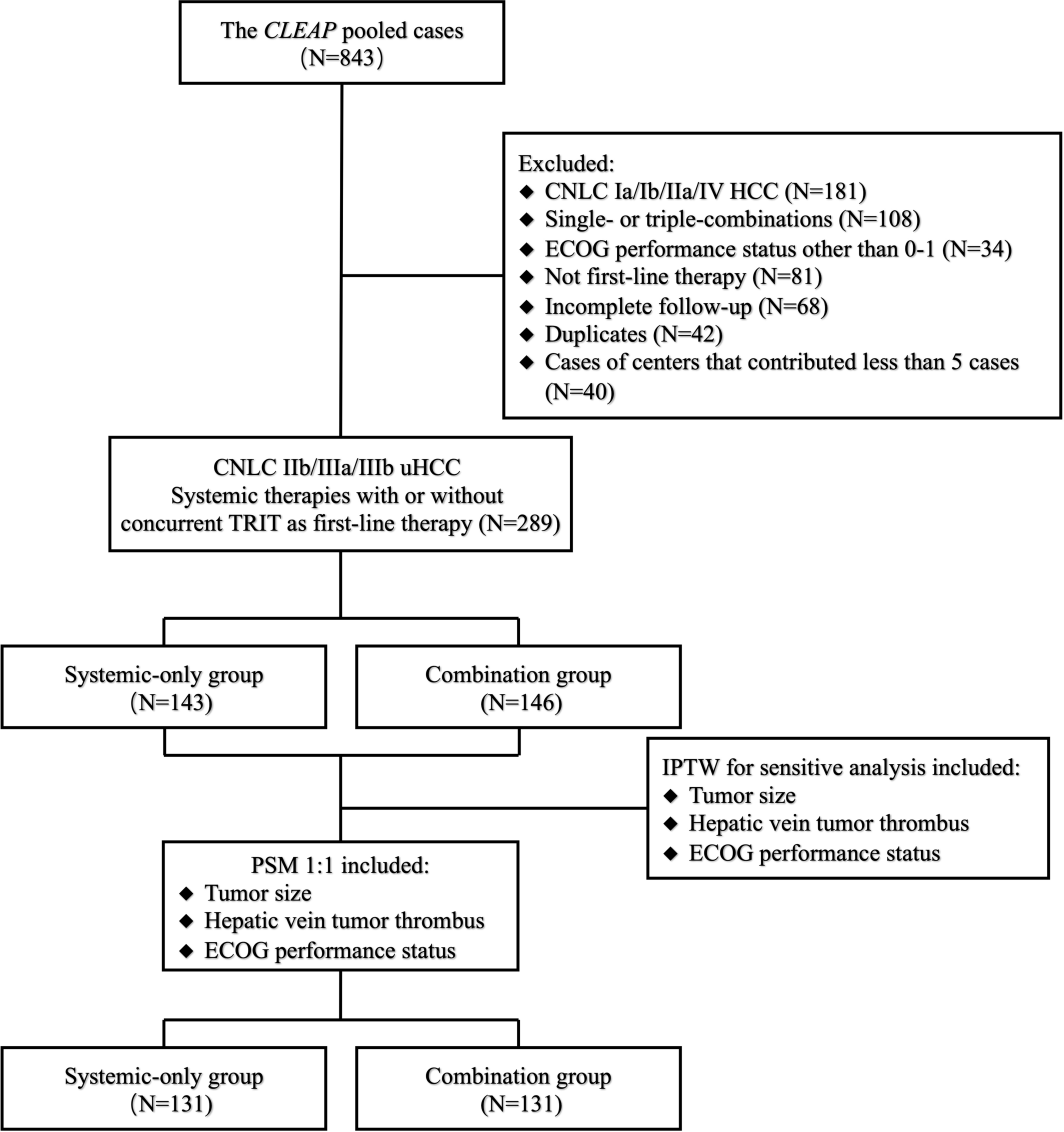
Patient disposition. CLEAP, China Liver Cancer Study Group Young Investigators; CNLC, China liver cancer; ECOG, Eastern Cooperative Oncology Group; uHCC, unresectable hepatocellular carcinoma; PSM, propensity score matching; IPTW, inverse probability of treatment weighting.

The analysis included 289 eligible patients treated with first-line systemic therapy with TKIs plus PD-1 antibodies, with or without concurrent TRIT, between 21 September 2018 and 26 April 2022. Patient disposition is shown in **Figure 1**. In the overall population, the median age was 54.00 years (IQR, 48.00 to 61.00), and the majority of patients were male (90.3%) (**Table 1**). Most patients had hepatitis B virus (HBV) infection (serum positivity for HBsAg, HBcAb, or HBeAb) as the etiology of uHCC (86.9%), Child-Pugh class A liver function (95.2%), and ECOG PS 0 (74.4%). Overall, 18.0, 46.4, and 35.6% of patients were diagnosed with CNLC stages IIb, IIIa, and IIIb, respectively.

**Table 1:** Baseline demographics and disease characteristics and outcomes of patients before and after PSM.

Characteristic	Overall	Before PSM				After PSM			
		Systemic-only group	Combination group	SMD	*P*-value	Systemic-only group	Combination group	SMD	*P*-value
	*N* = 289	*N* = 143	*N* = 146			*N* = 131	*N* = 131		
Sex, cases (%)				0.040	0.888			0.026	1.000
Male	261 (90.31)	130 (90.91)	131 (89.73)			119 (90.84)	118 (90.08)		
Female	28 (9.69)	13 (9.09)	15 (10.27)			12 (9.16)	13 (9.92)		
Age (years), median [IQR]	54.00 [48.00, 61.00]	54.00 [47.50, 60.00]	54.00 [48.00, 61.00]	0.021	0.938	54.00 [47.50, 60.50]	54.00 [48.50, 61.00]	0.027	0.867
Body mass index (kg/m^2^), median [IQR]	21.87 [19.91, 24.08]	21.80 [20.02, 23.91]	22.05 [19.92, 24.21]	0.216	0.926	21.80 [20.02, 23.91]	22.22 [19.94, 24.34]	0.223	0.958
Etiology of HCC, cases (%)				0.334	0.100			0.334	0.130
Hepatitis B virus	251 (86.85)	122 (85.31)	129 (88.36)			112 (85.50)	117 (89.31)		
Hepatitis C virus	3 (1.04)	0 (0.00)	3 (2.05)			0 (0.00)	2 (1.53)		
non-B non-C	35 (12.11)	21 (14.69)	14 (9.59)			19 (14.50)	12 (9.16)		
HBV-DNA copy numbers, median [IQR]	50.00 [30.00, 400.00]	50.00 [45.38, 499.25]	100.00 [12.00, 352.00]	0.301	0.346	50.00 [36.15, 457.50]	100.00 [3.00, 326.50]	0.330	0.483
Child-Pugh class, cases (%)				0.134	0.389			0.176	0.255
A	275 (95.16)	134 (93.71)	141 (96.58)			122 (93.13)	127 (96.95)		
B	14 (4.84)	9 (6.29)	5 (3.42)			9 (6.87)	4 (3.05)		
ECOG performance status, cases (%)				0.276	0.029			0.160	0.252
0	215 (74.39)	115 (80.42)	100 (68.49)			103 (78.63)	94 (71.76)		
1	74 (25.61)	28 (19.58)	46 (31.51)			28 (21.37)	37 (28.24)		
ALBI grade, cases (%)				0.137	0.508			0.102	0.772
Grade 1	139 (48.10)	73 (51.05)	66 (45.21)			65 (49.62)	61 (46.56)		
Grade 2	145 (50.17)	67 (46.85)	78 (53.42)			63 (48.09)	68 (51.91)		
Grade 3	5 (1.73)	3 (2.10)	2 (1.37)			3 (2.29)	2 (1.53)		
BCLC stage, cases (%)				0.104	0.469			0.021	1.000
B	45 (15.57)	25 (17.48)	20 (13.70)			19 (14.50)	19 (14.50)		
C	244 (84.43)	118 (82.52)	126 (86.30)			112 (85.50)	112 (85.50)		
China liver cancer stage, cases (%)				0.063	0.866			0.040	0.886
IIb	52 (17.99)	27 (18.88)	25 (17.12)			21 (16.03)	24 (18.32)		
IIIa	134 (46.37)	67 (46.85)	67 (45.89)			64 (48.85)	62 (47.33)		
IIIb	103 (35.64)	49 (34.27)	54 (36.99)			46 (35.11)	45 (34.35)		
Tumor size (mm), mean (SD)	88.05 (46.93)	78.09 (45.89)	98.59 (45.88)	0.447	0.001	84.46 (41.31)	90.70 (36.62)	0.160	0.197
Intrahepatic tumor number, cases (%)				0.063	0.866			0.040	0.886
1	134 (46.37)	67 (46.85)	67 (45.89)			64 (48.85)	62 (47.33)		
2–3	103 (35.64)	49 (34.27)	54 (36.99)			46 (35.11)	45 (34.35)		
≥ 4	52 (17.99)	27 (18.88)	25 (17.12)			21 (16.03)	24 (18.32)		
Presence of portal vein invasion, cases (%)	169 (58.48)	75 (52.45)	94 (64.38)	0.244	0.052	71 (54.20)	81 (61.83)	0.171	0.260
Presence of hepatic vein invasion, cases (%)	43 (14.88)	12 (8.39)	31 (21.23)	0.368	0.004	12 (9.16)	21 (16.03)	0.208	0.136
Presence of extrahepatic disease, cases (%)	109 (37.72)	50 (34.97)	59 (40.41)	0.113	0.404	48 (36.64)	48 (36.64)	< 0.001	1.000
Baseline AFP level (ng/ml), mean (SD)	12,668.76 (49,669.97)	19,200.18 (82,966.35)	22,366.19 (44,313.27)	0.048	0.703	20,331.47 (85,642.76)	20,461.84 (36,365.99)	0.002	0.987
Baseline PIVKA-II level (mAU/ml), mean (SD)	13,672.99 (24,364.87)	13,847.92 (23,270.02)	29,345.13 (1e+05)	0.208	0.106	14,129.49 (21,089.25)	27,529.41 (88,687.16)	0.088	0.094
Conversion to surgery, cases (%)	47 (16.26)	22 (15.38)	25 (17.12)	0.047	0.810	21 (16.03)	17 (12.98)	0.087	0.599
Objective response^*^, cases (%)				0.072	0.624			0.097	0.604
CR+PR	105 (36.33)	51 (35.66)	54 (36.99)			43 (32.82)	48 (36.64)		
Controlled disease^*^, cases (%)				0.078	0.593			0.100	0.499
CR+PR+SD	246 (85.1)	119 (83.22)	127 (86.99)			109 (83.21)	112 (85.50)		
Deaths for any reason, cases (%)	85 (29.41)	50 (34.97)	35 (23.97)	0.243	0.055	44 (33.59)	33 (25.19)	0.185	0.175

PSM, propensity score matching; SMD, standard mean difference; IQR, interquartile range; SD, standard deviation; HCC, hepatocellular carcinoma; HBV, hepatitis B virus; ECOG, eastern cooperative oncology group; ALBI, albumin-bilirubin grade, BCLC, Barcelona clinic liver cancer; AFP, alpha-fetoprotein; PIVKA-II, protein induced by vitamin K absence-II; CR, complete response; PR, partial response; SD, stable disease; RECIST response evaluation criteria in solid tumors.

^*^According to RECIST v1.1 criteria.

Although baseline variables were generally comparable between the two groups, the combination *versus* systemic-only group had significantly higher percentages of patients with ECOG PS 1 (31.5 *vs.* 19.6%, respectively; *P* = 0.029), hepatic vein invasion (21.2 *vs.* 8.4%, respectively; *P* = 0.004), and larger tumor size (98.6 ± 45.9 *vs.* 78.1 ± 45.9 mm, respectively; *P* = 0.001).

### Treatment overview

Patients received various oral TKIs, including lenvatinib (87.9%), apatinib (8.3%), and sorafenib (3.5%), and anti–PD-1 antibodies, including sintilimab (31.2%), camrelizumab (29.4%), toripalimab (21.1%), tislelizumab (9.0%), pembrolizumab (6.7%), and nivolumab (2.6%). TRIT (i.e., TACE and HAIC) was administered following digital subtraction angiography *via* right femoral artery puncture and catheterization. TACE agents were mainly epirubicin and platinum with lipiodized oil, while HAIC mostly comprised oxaliplatin, leucovorin, and fluorouracil on the first day, with maintenance of the fluorouracil infusion for 23h or 46h, according to standard local practice. Treatment was continued until PD, unacceptable toxicity, or curative hepatectomy. For patients with HBV infection, the oral antiviral agents entecavir or tenofovir were prescribed throughout anti-cancer treatment.

### Survival and tumor response

At the data cut-off of 19 August 2022, the median follow-up was 11.57 months (IQR, 6.73 to 20.33) for the overall population. A total of 85 (29.4%) patients had died, and the median OS for all patients was 34.33 months (95% confidence interval [CI], 23.00 months to not reached [NR]), while the median PFS was 12.27 months (95% CI, 9.27 months to 17.93 months).

**Figure 2:**
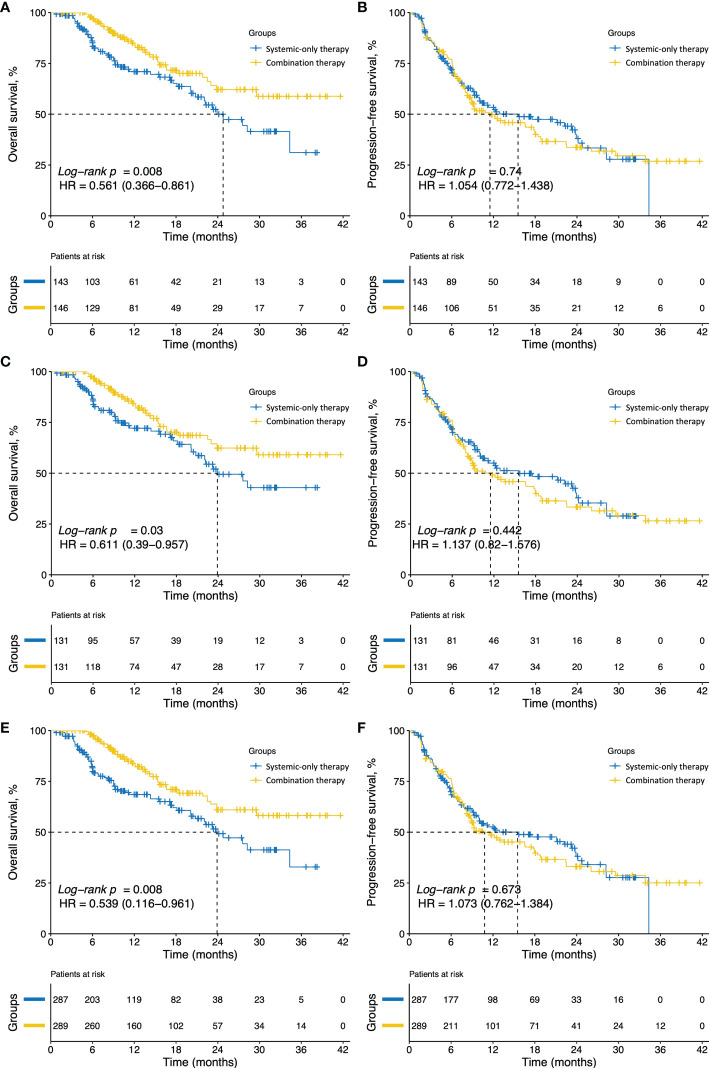
Kaplan–Meier curves of overall survival (OS) and progression-free survival (PFS) for patients in the different groups. **(A)** OS and **(B)** PFS of patients in the systemic-only group (*N* = 143) or combination group (*N* = 146) before PSM. **(C)** OS and **(D)** PFS for the systemic-only group (*N* = 131) and combination group (*N* = 131) after PSM. **(E)** OS and **(F)** PFS for patients in the systemic-only group (*N* = 287.37) and combination group (*N* = 289.04) weighted by IPTW. HR, hazard ratio; PSM, propensity score matching; IPTW, inverse probability of treatment weighting.

During follow-up, 35 patients (24.0%) in the combination group and 50 (34.8%) in the systemic-only group died. The median OS was significantly longer in the combination group compared with the systemic-only group [hazard ratio (HR), 0.561; 95% CI, 0.366 to 0.861; *P* = 0.008; **Figure 2A**], with 12-, 24-, and 36-month OS rates of 83.9, 62.1, and 58.8%, respectively, with combination therapy and 71.0, 50.0, and 31.1% with systemic-only therapy. However, the 12-, 24-, and 36-month PFS rates were comparable between the groups: 48.8, 33.7, and 26.9%, respectively, in the combination group and 53.4, 38.0, and 27.8% in the systemic-only group (HR, 1.054; 95% CI, 0.772 to 1.438; *P* = 0.740; **Figure 2B**).

**Figure 3:**
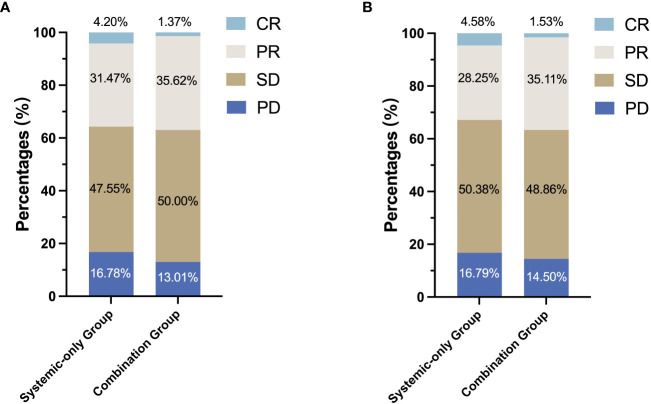
Tumor responses rates based on RECIST v1.1 in the systemic-only group and the combination group **(A)** before PSM and **(B)** after PSM. RECIST, Response Evaluation Criteria in Solid Tumors; PSM, propensity score matching; CR, complete response; PR, partial response; SD, stable disease; PD, progressive disease.

In the combination group, the best response was a CR in two patients, a PR in 52 patients, and SD in 73 patients, providing an ORR of 37.0%. The best response in the systemic-only group was a CR in six patients, a PR in 45 patients, and SD in 68 patients, giving an ORR of 32.9% (**Figure 3A**). Tumors in 25 patients (17.1%) in the combination group and 22 patients (15.4%) in the systemic-only group were successfully converted to become surgically resectable. Response and conversion rates did not differ significantly between the two groups (**Table 1**).

In multivariate analyses, combination therapy was significantly and independently associated with improved OS compared with systemic therapy (HR, 0.286; 95% CI, 0.100 to 0.813; *P* = 0.019). Furthermore, objective tumor response was significantly associated with increases in both OS (HR, 0.319; 95% CI, 0.133 to 0.762; *P* = 0.010) and PFS (HR 0.432; 95% CI, 0.219 to 0.851; *P* = 0.015) (**Table 2**).

**Table 2:** The Cox regression analysis for overall survival and progression free survival.

Characteristics	Overall survival				Progression-free survival			
	Univariate analysis	Multivariate analysis			Univariate analysis	Multivariate analysis		
	*P*-value	HR	95% CI	*P*-value	*P*-value	HR	95% CI	*P*-value
Sex (male *vs.* female)	0.577				0.750			
Age (≥ 60 *vs.* < 60 years)	0.506				0.094	1.206	0.598–2.433	0.602
Body mass index (≥ 22 kg/m^2^ *vs.* < 22 kg/m^2^)	0.614				0.776			
Etiology of HCC (HBV *vs.* HCV *vs.* non-viral)	1.000				0.734			
China liver cancer stage (IIa *vs.* IIIa *vs.* IIIb)	0.574	0.196	0.032–1.206	0.079	0.026	0.440	0.114–1.694	0.232
Presence of portal vein invasion (yes *vs.* no)	0.214				0.911			
Presence of hepatic vein invasion (yes *vs.* no)	0.264				0.718			
Baseline AFP (≥ 400 *vs.* < 400 ng/ml)	0.660				0.325			
Baseline PIVKA-II (< 1000 *vs.* ≥ 1000 mAU/ml)	0.997				0.531			
Intrahepatic tumor number (1-3 *vs.* ≥ 4)	0.921				0.034	1.151	0.196–6.745	0.876
Objective tumor response per RECIST v1.1 (responders *vs.* non-responders)	< 0.001	0.319	0.133–0.762	0.010	< 0.001	0.432	0.219–0.851	0.015
Concurrent TRIT (yes *vs.* no)	0.008	0.286	0.100–0.813	0.019	0.741	1.121	0.502–2.506	0.781

HR, hazard ratio; CI, confidence interval; HCC, hepatocellular carcinoma; HBV, hepatitis B virus; HCV, hepatitis C virus; AFP, alpha-fetoprotein; PIVKA-II, protein induced by vitamin K absence-II; RECIST, response evaluation criteria in solid tumours; TRIT, transcatheter intra-arterial therapies.

### Survival analysis and tumor response after adjustment

After 1:1 PSM for tumor size, hepatic vein invasion and ECOG PS, all baseline variables were comparable across the two treatment groups (all *P* > 0.050) (**Table 1**). The distributions of imbalanced variables before and after PSM adjustment are shown in the **Supplementary Materials** (**Supplementary Figure S1**). Of note, following PSM adjustment, OS in the combination group remained significantly longer, with 12-, 24-, and 36-month OS rates of 83.2, 62.4, and 59.1%, compared with 72.2, 49.5, and 42.9% in the systemic-only group (HR, 0.612; 95% CI, 0.390 to 0.957; *P* = 0.031; **Figure 2C**). PFS was not significantly different between the two groups after PSM adjustment, with 12-, 24-, and 36-month PFS rates of 48.0, 33.3, and 36.6%, respectively, for the combination group and 55.0%, 37.9% and 26.6% for the systemic-only group (HR, 1.137; 95% CI, 0.82 to 1.576; *P* = 0.442; **Figure 2D**). Furthermore, the ORR was not significantly different between the two groups after PSM adjustment (36.6% *vs.* 32.8%, *P* = 0.604; **Figure 3B**).

After IPTW (**Supplementary Figure S2**), baseline tumor size, prevalence of hepatic vein invasion and ECOG PS were similar between the two groups (*P* > 0.050) (**Supplementary Table S1**). The OS remained significantly longer in the combination group with 12-, 24- and 36-month OS rates of 83.1, 61.0, and 58.2%, respectively, compared with 68.5, 49.3, and 32.9% for the systemic-only group (HR, 0.539; 95% CI, 0.116 to 0.961; *P* = 0.008; **Figure 2E**). Consistent results were obtained in analyses of PFS and ORR before and after IPTW adjustment (**Figure 2F**; **Supplementary Table S1**, respectively).

### Overall survival subgroup analysis

**Figure 4:**
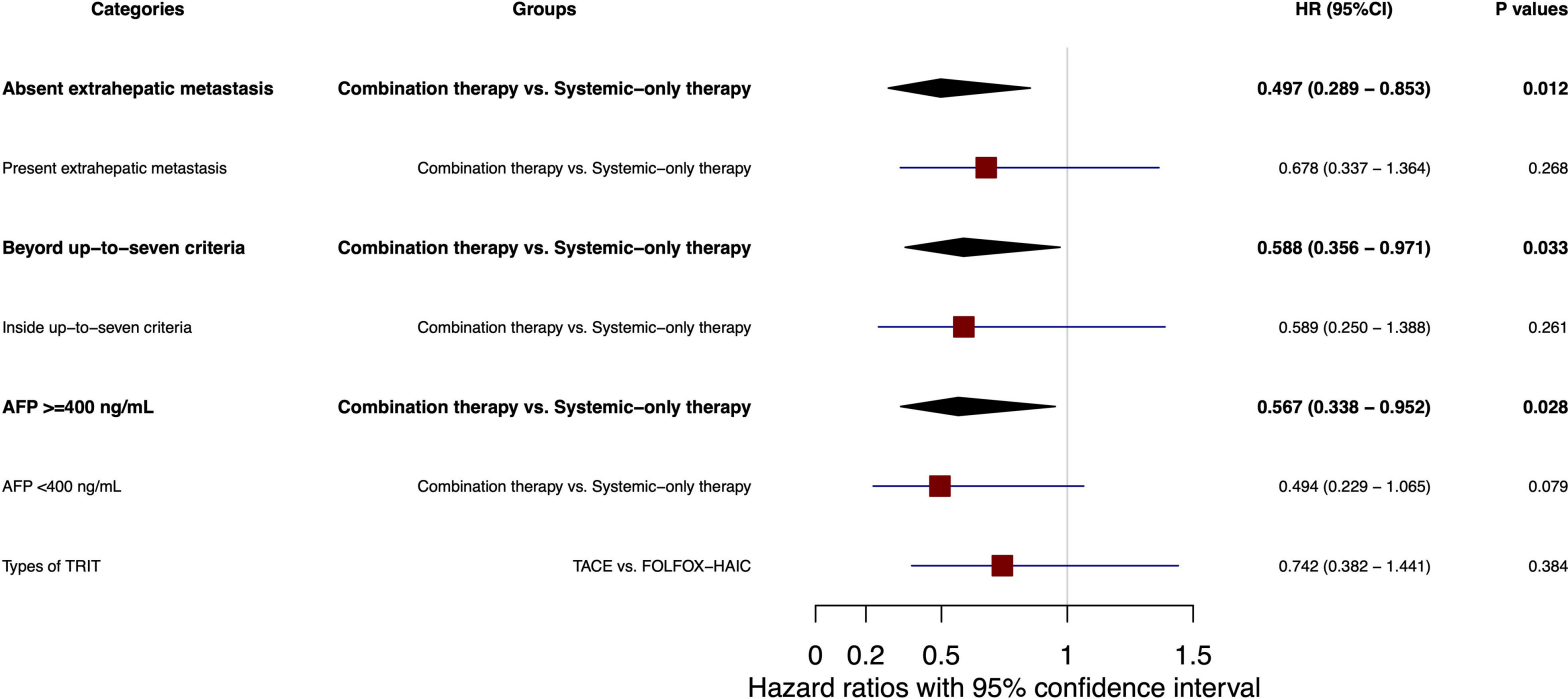
Forest plot of subgroup analysis for overall survival. AFP, alpha-fetoprotein; TRIT, transcatheter intra-arterial therapies; TACE, transarterial chemoembolization; HAIC, hepatic arterial infusion chemotherapy; HR, hazard ratio; 95% CI, confidence interval.

In subgroup analyses, patients without extrahepatic metastasis significantly benefited from combination *versus* systemic therapy (OS HR, 0.497; 95% CI, 0.289 to 0.853; *P* = 0.012; **Figure 4**), whereas no difference was observed in patients with extrahepatic metastasis (OS HR, 0.278; 95% CI, 0.337 to 1.364; *P* = 0.268; **Figure 4**). Interestingly, a significantly longer OS was demonstrated with combination *versus* systemic therapy among patients with liver lesions exceeding the up-to-seven criteria (HR, 0.588; 95% CI, 0.356 to 0.971; *P* = 0.033; **Figure 4**), while no significant difference was observed among patients within these criteria (HR, 0.261; 95% CI, 0.250 to 1.388; *P* = 0.261; **Figure 4**). Additionally, OS was significantly longer in the combination group *versus* the systemic-only group for patients with baseline AFP ≥ 400 ng/ml (HR, 0.567; 95% CI, 0.338 to 0.952; *P* = 0.028; **Figure 4**), but not in those with AFP < 400 ng/ml (HR, 0.494; 95% CI, 0.229 to 1.065; *P* = 0.079; **Figure 4**). OS was comparable between patients who received TACE or HAIC as their concurrent TRIT with systemic therapy (HR, 0.742; 95% CI, 0.382 to 1.441; *P* = 0.384; **Figure 4**).

### Safety

No treatment-related deaths occurred in either treatment group. Severe (i.e., grade 3/4) TRAEs were reported in 16 patients (11.0%) in the combination group and 17 patients (11.9%) in the systemic-only group (*P* = 0.223). There was no significant difference between the two groups in the types of severe TRAEs reported (*P* = 0.139) (**Table 3**).

**Table 3:** Overall summary of grades 3–4 treatment-related adverse events.

	Systemic-only group	Combination group	Total	*P*-value
Patients number, *n*	143	146	289	
Patients with grades 3–4 TRAEs, *n* (%)	17 (11.89)	16 (10.96)	33 (11.42)	0.223
Categories of grades 3–4 TRAEs, *n* (%)^#^				0.139
Vomit	1 (0.70)	2 (1.37)	3 (1.04)	
Hand-foot syndrome	3 (2.10)	2 (1.37)	5 (1.73)	
Hypoleukemia	4 (2.80)	3 (2.05)	7 (2.42)	
Liver damage	2 (1.40)	3 (2.05)	5 (1.73)	
Kidney damage	2 (1.40)	2 (1.37)	4 (1.38)	
Infection	2 (1.40)	2 (1.37)	4 (1.38)	
Hypoplateletemia	1 (0.70)	0 (0.00)	1 (0.35)	
Hypertension	2 (1.40)	2 (1.37)	4 (1.38)	

TRAEs, treatment-related adverse events.

^#^Patients may be counted in more than one category.

## Discussion

To our knowledge, this is the first real-world, multi-center study to compare the efficacy and safety of TKIs plus PD-1 antibodies, with or without concurrent TRIT, for the first-line treatment of patients with uHCC. Patients in the combination group demonstrated significantly superior OS compared with systemic therapy alone in unadjusted analyses, as well as following PSM and IPTW adjustment. Multivariate analyses revealed that concurrent TRIT was significantly and independently associated with longer OS in this cohort of patients with uHCC of CNLC stages IIb to IIIb.

Therapeutic options for uHCC have evolved dramatically in recent years. Following the demonstration of a significant benefit of lenvatinib *versus* sorafenib as first-line therapy for advanced HCC in the REFLECT study, several newer TKIs and PD-1 antibodies, alone or in combination, have been recommended in the first-line setting (6, 8, 26, 27). Following the failure of studies of single-agent PD-1 antibodies to show improvements in tumor control and initial skepticism regarding combination regimens, the Imbrave150 study demonstrated that atezolizumab plus bevacizumab increased 12-month OS by 12.6% compared with sorafenib, with a similar incidence of adverse events between groups (8). Moreover, in the HIMALAYA study, tremelimumab, an anti-cytotoxic T-lymphocyte-associated antigen-4 monoclonal antibody, combined with durvalumab, an anti-programmed death ligand-1 monoclonal antibody, prolonged median OS by 3.0 months compared with sorafenib, in patients with uHCC (26). More recently, the phase III LEAP-002 study of first-line lenvatinib plus pembrolizumab, compared with lenvatinib monotherapy, achieved the longest median OS (21.2 months) for dual therapy ever reported in the treatment of advanced HCC, further supporting the concept of combining systemic therapies in patients ineligible for curative therapy (27).

HCC is a hypervascular tumor that derives most of its blood supply from the hepatic artery. Accordingly, TRIT, including TACE and FOLFOX-HAIC, has been shown to facilitate local tumor control and provide long-term clinical benefits (28). TACE with cisplatin or doxorubicin has demonstrated favorable OS in the treatment of uHCC and is recommended for asymptomatic, large, or multifocal HCC without macrovascular invasion or extrahepatic metastasis (3, 29, 30). Additionally, several recent randomized clinical trials have shown FOLFOX-HAIC is another potent TRIT that improves survival outcomes compared with the guideline-recommended TACE or sorafenib in the treatment of large and advanced HCC tumors and have enabled patient eligibility criteria for TRIT to be enriched (5, 31). For example, in patients with large, unresectable HCC tumors exceeding 7 cm, FOLFOX-HAIC prolonged the median OS by 7.0 months compared with TACE (HR, 0.580; 95% CI, 0.450 to 0.750; *P* < 0.001) with a lower incidence of adverse events (*P* = 0.030) (5).

Given the encouraging efficacy and acceptable safety of TRIT in uHCC treatment, the administration of TRIT alongside systemic therapy has the potential to enhance liver lesion control with manageable safety. Indeed, the LAUNCH trial demonstrated that first-line lenvatinib plus TACE significantly improved median OS by 6.3 months compared with lenvatinib alone (*P* < 0.001), with a pronounced benefit observed in patients with tumor thrombosis (32). Another study found that sorafenib plus FOLFOX-HAIC provided a median OS of 13.37 months, which is significantly longer than with sorafenib monotherapy, at 7.13 months (*P* < 0.001) (13). Moreover, the potential to further evolve combination approaches was illustrated in a single-arm study in which lenvatinib plus sintilimab plus TACE led to a median OS of 23.6 months (95% CI, 22.2 to 25.0 months) in patients with uHCC (33). Nonetheless, evidence regarding the administration of both TKIs and PD-1 antibodies in combination with TRIT is limited.

While a previous small-scale, single-center study has shown the superiority of combination therapy with a TKI, ICI and TRIT over systemic therapy (34), the present study provides the first multi-center, head-to-head comparison of OS between dual systemic therapy with TKIs and PD-1 antibodies, with or without TRIT, in patients with uHCC. After comprehensive analyses incorporating PSM and IPTW adjustment to account for imbalances in patient characteristics, combination treatment consistently demonstrated an OS benefit over systemic-only therapy, while PFS and the incidence of TRAEs were comparable between groups. Interestingly, patients in the combination group had larger tumors, worse ECOG PS, and more hepatic vein invasion than patients in the systemic-only group. This may reflect a tendency for physicians to administer concurrent TRIT in patients with higher liver tumor burden, with the goal of improving primary liver lesion control to preserve liver function and so prolong survival. Patients with higher intrahepatic tumor burden tended to rapid progression, presenting originally shorter PFS and lower ORR. The concurrent TRIT could help to fix the unfavorable to some extent, resulting in similar PFS and ORR of the two groups. The lack of a significant difference between groups in PFS may also be partly explained by the generally shorter follow-up interval (4–6 weeks) between assessments in patients who received TRIT than those who received systemic therapy alone (6–8 weeks).

Our subgroup analyses indicated that the OS benefit from concurrent TRIT was derived by patients with a greater local tumor burden, including those whose liver lesions exceeded the up-to-seven criteria, and those without extrahepatic metastases. In these patients with aggressive liver tumors, improvement in local tumor control with concurrent TRIT may better preserve liver function, which could have a critical impact on OS in this setting. Although FOLFOX-HAIC was reported to improve OS *versus* TACE in a randomized phase III study in patients with large uHCC tumors (5), we found no survival difference between patients who received TACE or FOLFOX-HAIC as concurrent TRIT with dual systemic therapy. In combination with systemic therapy, both TACE and HAIC are reported to improve disease control and prognosis in patients with uHCC in single-center or retrospective studies (35–37). The results of the present study therefore demonstrate that the combination of TKIs plus PD-1 antibodies with concurrent TRIT is a feasible strategy that holds promise for the treatment of patients with uHCC.

This study has several limitations. First, the combinations of TKIs and PD-1 antibodies are not considered standard regimens for the first-line treatment of uHCC in Western countries. Second, since bevacizumab plus atezolizumab was not approved in China until the end of 2020, this regimen was infrequently used in the present dataset (11, 38). Third, a lack of independent tumor assessments or standardization of TRIT protocols between different centers may have impacted the reliability of PFS and ORR estimates in this retrospective, multi-center study. Finally, the study included predominantly patients with HBV-related HCC, who are known to benefit more from immune-based therapies than those with HCC of other eiologies (39). Thus, our findings should be independently verified in broader populations, including patients outside China.

## Conclusions

In conclusion, TKIs plus PD-1 antibodies combined with concurrent TRIT were associated with superior OS compared with dual systemic therapy alone in Chinese patients with uHCC, especially in those with a high-intrahepatic tumor load and no extrahepatic metastasis. Prospective studies with a larger sample size and longer follow-up are required to validate these findings.

## Data availability statement

The original contributions presented in the study are included in the article/**Supplementary Material**. Further inquiries can be directed to the corresponding authors.

## Ethics statement

The studies involving human participants were reviewed and approved by Institutional Review Board of Sun Yat-sen University Cancer Center and Zhongshan Hospital, Fudan University (Approval No. B2022-301-01 and B20202-195R, respectively). The patients/participants provided their written informed consent to participate in this study.

## Author contributions

YP, XZ, HS and LX designed experiments and drafted the manuscript. JL, JZ, WZ, SS, RJ, HL, FY, KH, DX, YZ, LZ, BCX, ZC, YC, YYZ, XL, MK, TS, BDX and KW collected the cases. XZ, HS and LX approved the final version. The China Liver Cancer Study Group Young Investigators (CLEAP) provide the platform for data maintenance. All authors contributed to the article and approved the submitted version.
