# Supplementary material for: Systemic therapy with or without transcatheter intra-arterial therapies for unresectable hepatocellular carcinoma: a real-world, multi-center study
Source: Front Immunol. 2023 Apr 26;14:1138355. doi: 10.3389/fimmu.2023.1138355 (PMC10169746; doi:10.3389/fimmu.2023.1138355)
Supplement: Supplementary file 1 [file DataSheet_1.pdf]

Table S1. Weighted baseline characteristics of patients in the two groups before and after IPTW

Characteristic	Unweighted				Weighted			
	Systemic-only group	Combination group	SMD	*P* value	Systemic-only group	Combination group	SMD	*P* value
	N=143	N=146			N=287.37	N=289.04		
Tumor size (mm), mean (SD)	79.27 (43.18)	96.64 (41.59)	0.410	0.001	88.22 (44.94)	88.59 (41.35)	0.008	0.948
Presence of hepatic vein invasion, cases (%)	12 (8.39)	31 (21.23)	0.368	0.004	39.5 (13.74)	42.7 (14.77)	0.029	0.822
ECOG performance status score 1, cases (%)	28 (19.58)	46 (31.51)	0.276	0.029	69.9 (24.32)	72.8 (25.18)	0.020	0.873
Objective response per RECIST v1.1, cases (%)	47 (32.86)	53 (36.30)	0.072	0.624	95.8 (33.33)	108.0 (37.37)	0.084	0.499

IPTW, inverse probability of treatment weighting; ECOG, eastern cooperative oncology group; SD, standard deviation; SMD, standard mean difference; RECIST, response evaluation criteria in solid tumours.

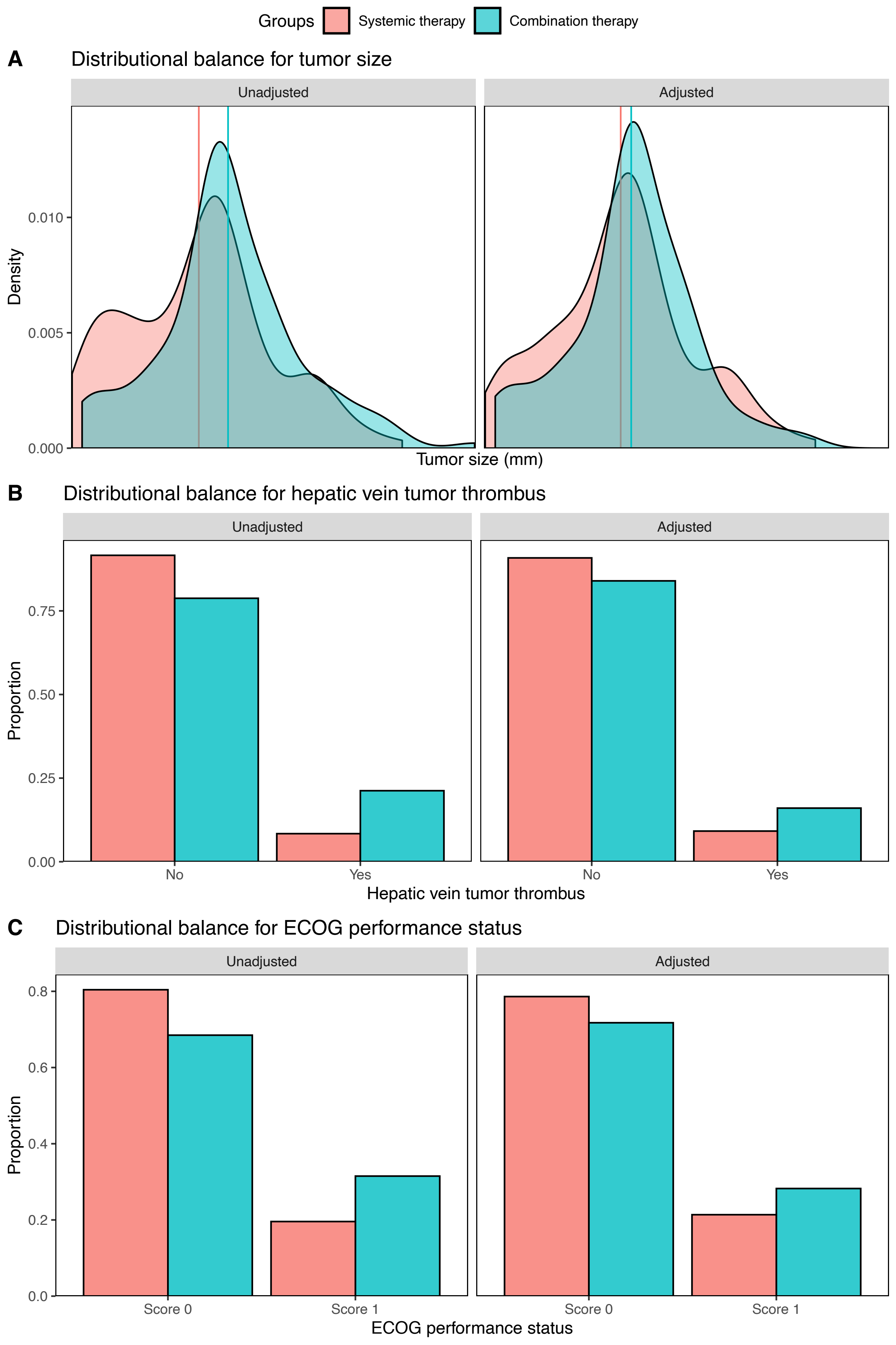

Figure S1. The distributions of tumor size (**A**), hepatic vein invasion (**B**), and ECOG performance status (**C**) before and after PSM. ECOG, Eastern Cooperative Oncology Group; PSM, propensity score matching.

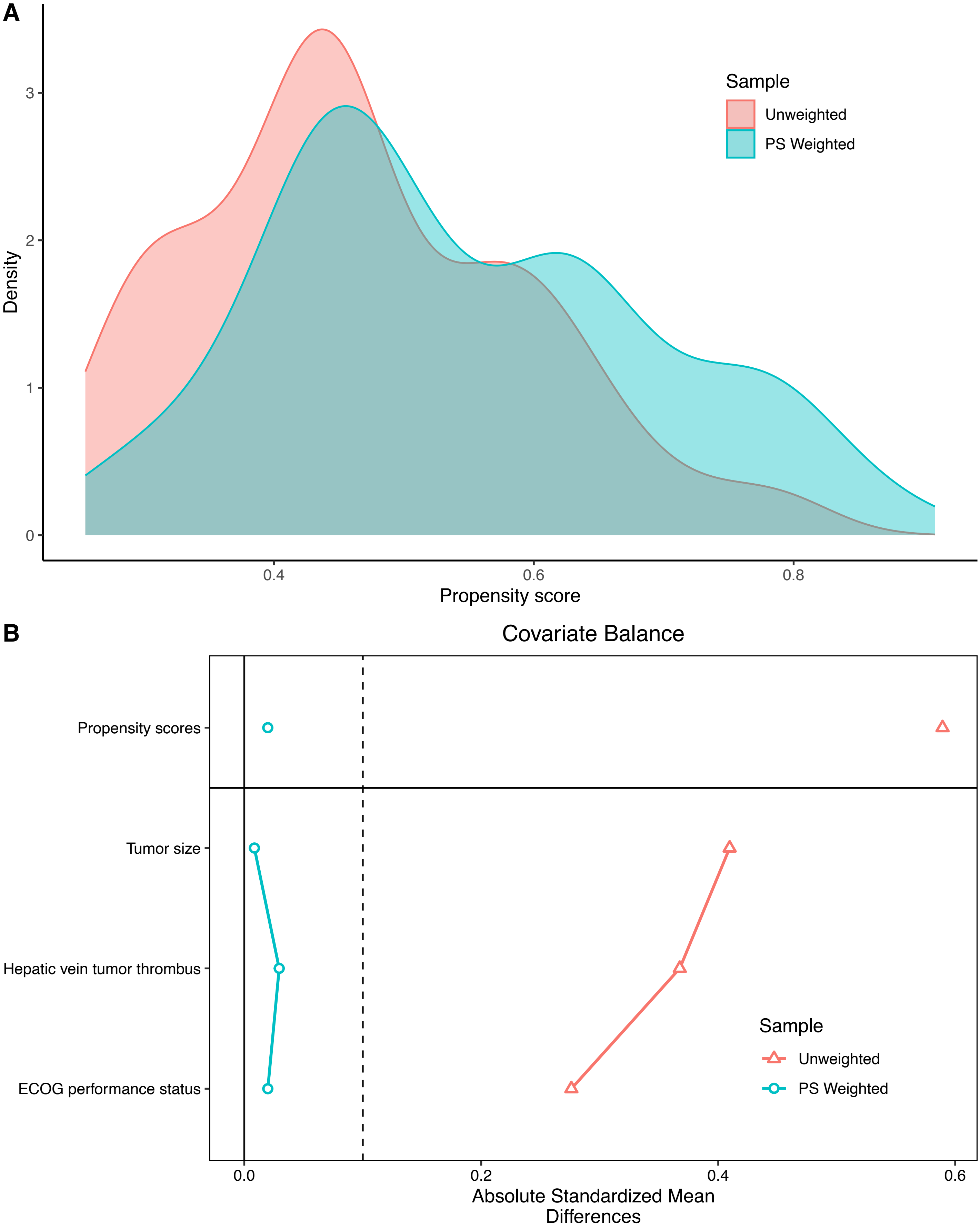

Figure S2. The distributions of the propensity score for unweighted and weighted cohorts by IPTW (A), and the distributions of covariate balance for unbalanced variables before and after introduced to IPTW (B). IPTW, inverse probability treatment weighting; PS, propensity score; ECOG, Eastern Cooperative Oncology Group.
